# Exploring fashion designers’ acceptance of AIGC: A dual-pathway analysis from the stimulus–organism–response perspective

**DOI:** 10.1371/journal.pone.0356536

**Published:** 2026-08-17

**Authors:** Tingting Ma, Mengyun Yang

**Affiliations:** 1 School of Fashion Design, Jiangxi Institute of Fashion Technology, Nanchang, China; 2 Nanchang Key Laboratory of Artificial Intelligence Manufacturing for Light Industrial Products, Jiangxi Institute of Fashion Technology, Nanchang, Jiangxi, China; Sichuan University, CHINA

## Abstract

Artificial Intelligence Generated Content (AIGC) is increasingly used in creative design. Understanding fashion designers’ willingness to adopt these tools has therefore become important for both research and practice. Drawing on the Stimulus–Organism–Response (SOR) model, this study integrates Self-Determination Theory (SDT) with perceived risk, social influence, and facilitating conditions. It examines how these contextual stimuli shape designers’ basic psychological need satisfaction and behavioral intention. We analyzed 318 valid responses with complete data for all 21 measurement items from Chinese fashion-design practitioners using partial least squares structural equation modeling (PLS-SEM). Perceived risk negatively predicted autonomy, competence, and relatedness, whereas social influence and facilitating conditions positively predicted these organismic states. Autonomy, competence, and relatedness each positively predicted behavioral intention, with competence showing the largest coefficient (β = 0.520, p < 0.001). Bootstrapped analyses confirmed all nine specific indirect effects from the three stimuli to behavioral intention through the three psychological needs. These findings clarify how SOR and SDT jointly explain technology adoption among fashion designers. They also provide practical guidance for copyright governance, prompt training, and collaboration between designers and AI.

## 1 Introduction

With the rapid development of Artificial Intelligence Generated Content (AIGC) technology, the creative design industry is undergoing a transformation. Especially in the field of fashion design, AIGC technologies, such as Stable Diffusion and Midjourney, have been gradually embedded into pattern generation, style modeling, inspiration and other aspects, greatly enhancing design efficiency and freedom of expression. These technologies not only provide designers with more diversified creative tools, but also inject new momentum into the digital transformation of the apparel industry chain. Over the next three to five years, generative AI could add up to $150 billion at a conservative estimate, adding up to $275 billion to the operating profits of the apparel, fashion, and luxury industries [1]. However, despite its significant technological potential, AIGC adoption among fashion designers remains limited in practice. The low acceptance of AIGC by some designers [2] hides more complex psychological motivations and external environmental factors, which are worth exploring in depth.

Research on AIGC adoption has frequently drawn on the Technology Acceptance Model (TAM) [3], which emphasizes the influence of perceived usefulness and perceived ease of use. These evaluations are important, but a purely functional account is insufficient for fashion design, where adoption also depends on whether designers retain volition, feel capable of controlling the tool, and experience professional connection and support. SDT provides a basis for examining these organismic experiences through autonomy, competence, and relatedness [4,5]. These constructs represent basic psychological needs or need-satisfaction states; they are not direct measures of intrinsic motivation. SDT also does not by itself specify how negative and enabling features of the external environment enter the adoption process. Perceived Risk concerning originality, copyright, output controllability, and professional replacement therefore needs to be examined together with social and resource-based support.

To address this gap, the present study uses the SOR framework to organize a model in which perceived risk (PR), social influence (SI), and facilitating conditions (FC) act as external stimuli; autonomy (AUT), competence (COMP), and relatedness (REL) represent organismic basic-need-satisfaction states; and behavioral intention (BI) represents the response. PR captures uncertainty and possible losses associated with AIGC use. SI captures peer norms and organizational expectations, as conceptualized in the UTAUT framework [6]. FC captures the technical infrastructure, training, and assistance that reduce access barriers [7]. This mapping gives each construct a distinct theoretical role: SOR supplies the stimulus-to-organism-to-response structure, SDT specifies the organismic states, and technology-acceptance and perceived-risk research motivates the external predictors.

Using survey data from Chinese fashion-design practitioners, this study tests the direct paths from the three stimuli to autonomy, competence, and relatedness; the paths from these organismic states to behavioral intention; and the corresponding specific indirect effects. The study contributes by distinguishing basic psychological need satisfaction from intrinsic motivation, empirically testing the complete stimulus–organism–response transmission process, and identifying competence as the principal organismic predictor of AIGC acceptance. The findings also inform concrete practices for copyright governance, prompt-based training, and human-centered designer–AI collaboration.

## 2 Literature review and conceptual development

### 2.1 The rise and challenges of AIGC in fashion design

AIGC is one of the important breakthroughs in AI technology in recent years. By integrating basic algorithms such as Generative Adversarial Networks, Diffusion Models, and Transformer Architecture, the AIGC system is able to realize cross-modal and efficient content generation, including automatic creation of images, texts, videos, and 3D models. In this context, AIGC is gradually moving from a general-purpose content generation tool to deeply embedded applications in various industries, especially in creative industries such as visual design, media, advertising and fashion design [8].

In the field of fashion design, AIGC is widely used in many aspects of garment pattern generation, style simulation and effect creation. Designers can quickly generate conceptual drawings through text input, enhance design efficiency with AI, and optimize the ability to capture inspiration and visual expression [9]. In a study by Zhang & Liu [10], the findings suggest that AI tools can help fashion designers create visually expressive garments and ready-to-wear that meet the designers’ design criteria and consumers’ needs. AIGC shortens the time from concept to presentation and reduces the cost of product development compared to traditional rendering methods.

However, AIGC applications in fashion design also create practical concerns. Designers may question originality, copyright ownership, output controllability, stylistic convergence, and the possible devaluation of professional roles [11,12]. These concerns can weaken trust in AIGC and shape whether designers experience autonomy, competence, and relatedness when incorporating the technology into creative work. Accordingly, adoption depends not only on perceived functional advantages but also on basic psychological need satisfaction and perceptions of technological uncertainty. A multidimensional account of external context and organismic experience is therefore needed to explain designers’ acceptance decisions and inform the responsible integration of AIGC into fashion-design practice.

### 2.2 Theory of stimulus-organism-response (SOR)

The SOR model was originally proposed by environmental psychologists Mehrabian and Russell to explain how external environmental stimuli can trigger changes in an individual's internal mental state, which in turn leads to behavioral responses [13]. The model suggests that stimuli (S) are external environmental factors that trigger changes in the internal cognitive or affective state of an organism (O), resulting in a corresponding response (R). In recent years, the SOR model has been widely used in the fields of consumer behavior, e-commerce, and acceptance of virtual reality and artificial intelligence technologies [14–16]. With its clear structure, it can systematically show how the external environment influences individual behavior through psychological mechanisms, and is an important theoretical tool for studying the mechanism of user technology adoption.

The SOR framework has been explored by some scholars in AIGC research. For example, Gu et al. [17] explored consumers’ acceptance behavior of AI-generated advertisements based on the SOR model, and found that external advertisement features (e.g., presentation style, content quality) affect users’ cognitive and emotional states, which in turn influence their acceptance willingness. Zhou and Lu [18] introduced the SOR model in a digital library context and analyzed the mechanism of users’ trust in AIGC content. Although the application of SOR in the creative industry is relatively rare, Wang and Chen [19] attempted to integrate it with TAM and TPB to study graphic, product, and interaction design practitioners’ attitudes and willingness to adopt the AIGC tool, suggesting that the SOR structure is also applicable to modeling the psychology and behavior of creative workers. However, the above studies did not cover the fashion designer population, an industry with fewer studies on AIGC adoption.

In terms of motivational factors, some studies have incorporated psychological variables related to motivation, although they do not directly invoke the theory of motivation. For example, Wang and Chen [19] incorporated self-efficacy and subjective norms into the SOR model, which represent individual confidence and social pressure, respectively, and these factors can influence users’ motivation to adopt. Meanwhile, Liu et al. [20] introduced the emotional motivation variable “awe” based on the SOR model and found that positive awe (inspiration) enhanced users’ persuasive perceptions of AIGC content, while negative awe (threat) weakened this perception. These studies show that the SOR model is highly flexible and can be used in conjunction with perceived risk (e.g., trust, privacy and security) and motivation-related variables (e.g., emotion, self-efficacy) to expand its explanatory power. Although there are still few studies that directly integrate a complete motivation theory (e.g., SDT) into the SOR model, existing empirical evidence has validated that the framework can effectively cover the process of users’ psychological responses to AIGC technology.

### 2.3 Stimulus factors

#### 2.3.1 Perceived risk (PR).

PR is defined as an individual's subjective perception of uncertainty about the possibility of suffering losses or adverse consequences when using a product or technology [21]. It is widely regarded as a key variable inhibiting users’ behavioral intention in technology adoption studies, especially in digital finance, online healthcare and AI scenarios, and its negative impact has been confirmed by a large number of empirical studies [22–24].

From the perspective of the SOR framework, Perceived risk can be conceptualized as an external stimulus that influences individuals’ internal cognitive or affective states and ultimately shapes their behavioral responses. Empirical evidence supports this positioning. For example, in the context of mobile health services, perceived risk has been shown to reduce continuance intention by weakening users’ trust in the platform [25]. Accordingly, treating perceived risk as an external stimulus is appropriate for explaining AIGC adoption in creative industries because it captures psychological responses to possible loss and uncertainty and clarifies how those responses may influence adoption decisions.

Perceived risk may affect behavioral intention indirectly by frustrating basic psychological need satisfaction. When risk is high, users may experience less control over technology use, which can reduce autonomy [26]. Uncertainty about operating AIGC can also reduce confidence in completing design tasks, weakening perceived competence. Concerns about originality, professional replacement, or unsafe outputs may further discourage interaction with other designers through the technology, weakening relatedness. Thus, perceived risk is expected to reduce all three organismic need-satisfaction states. This reasoning follows SDT, which identifies basic-need satisfaction as a condition for self-regulation [27].

Based on the above theoretical reasoning and empirical support, the following hypotheses are proposed:

H1a: Perceived risk negatively influences fashion designers’ perceived autonomy.
H1b: Perceived risk negatively influences fashion designers’ perceived competence.
H1c: Perceived risk negatively influences fashion designers’ perceived relatedness.

#### 2.3.2 Social influence (SI).

SI refers to an individual's decision making that is influenced by the perceptions of others or the norms of a social group [6]. In the field of technology acceptance, social influence has been widely validated as one of the key variables in predicting behavioral intentions, especially when users lack familiarity or information asymmetry with the emerging technology, the guiding role of this external environmental factor is particularly significant [28]. Its mechanism of action includes both normative influences (individuals’ behavior due to catering to group expectations) and informational influences (individuals’ acquisition of knowledge and modification of attitudes through the experiences of others) [29].

In the context of creative designers’ AIGC use, social influence can support basic psychological need satisfaction through several routes. Recognition from colleagues, organizations, or professional communities can strengthen designers’ sense that AIGC use is self-endorsed, supporting autonomy. Observing others’ workflows and receiving prompt or tool guidance can lower the learning threshold and strengthen competence. Participation in AIGC-oriented groups and collaborative projects can create professional belonging and strengthen relatedness [4].

SDT proposes that environments supporting autonomy, competence, and relatedness facilitate more self-determined regulation and sustained engagement [27]. In this study, AUT, COMP, and REL are measured as separate organismic need-satisfaction constructs; intrinsic motivation is not measured directly. We therefore interpret the proposed paths as effects of need satisfaction on behavioral intention, rather than as a direct test of intrinsic motivation. Consistent with the SOR framework, no direct path from SI to BI is hypothesized because the model specifies transmission through organismic states.

H2a: Social influences positively affect fashion designers’ sense of autonomy.

H2b: Social influences positively affect fashion designers’ sense of competence.

H2c: Social influences positively affect fashion designers’ sense of relatedness.

#### 2.3.3 Facilitating conditions (FC).

In technology adoption research, FC are defined as the availability of appropriate organizational and technical support when individuals perceive that they are using a system [6]. In AIGC-assisted fashion design, FC include suitable hardware and software, operational training, coworker assistance, and system stability. These resources can support autonomy by giving designers meaningful control over tool use, competence by helping them master prompt-based and iterative workflows, and relatedness by enabling collaboration and experience sharing [30–32].

FC therefore represents both an external resource and a context that can support basic psychological need satisfaction. When designers can obtain technical help, training, and peer support, they have more opportunities to explore AIGC voluntarily, master its functions, and participate in collaborative design work. Based on this reasoning, the following hypotheses are proposed:

H3a: Facilitating conditions positively influence fashion designers’ perceived autonomy in AIGC use.

H3b: Facilitating conditions positively influence fashion designers’ perceived competence in AIGC use.

H3c: Facilitating conditions positively influence fashion designers’ perceived relatedness in AIGC use.

### 2.4 Organismic states: Basic psychological needs

SDT identifies autonomy, competence, and relatedness as three basic psychological needs [33]. AUT refers to the feeling that one’s design choices are voluntary and self-endorsed. COMP refers to confidence in mastering the skills required to use AIGC and complete design tasks. REL refers to connection, recognition, and belonging within a community of designers. These constructs are distinct from intrinsic motivation: need satisfaction can support self-determined motivation, but the present questionnaire measures the three needs rather than motivation itself.

SDT predicts that social and technological environments supporting these needs promote internalized regulation and sustained engagement [34]. In creative work, autonomy supports authorship, competence supports confidence in iterative tool use, and relatedness supports exchange with peers and collaborators. The present model therefore places AUT, COMP, and REL in the organism layer and tests their separate effects on behavioral intention.

In the organism phase, this study examines whether designers feel autonomous when using AIGC, feel capable of operating the tools and completing design tasks, and experience connection and belonging with other designers through AIGC-related work. Prior evidence from AI-assisted design and other technology contexts shows that autonomy, competence, and relatedness can predict behavioral intention [35]. These findings support the use of SDT to specify the organismic mechanisms of AIGC adoption. Therefore, the following hypotheses are proposed in this study:

H4: Fashion designers’ perceived autonomy positively influences their behavioral intention toward AIGC use.

H5: Fashion designers’ perceived competence positively influences their behavioral intention toward AIGC use.

H6: Fashion designers’ perceived relatedness positively influences their behavioral intention toward AIGC use.

### 2.5 Conceptual model construction

**Fig 1:**
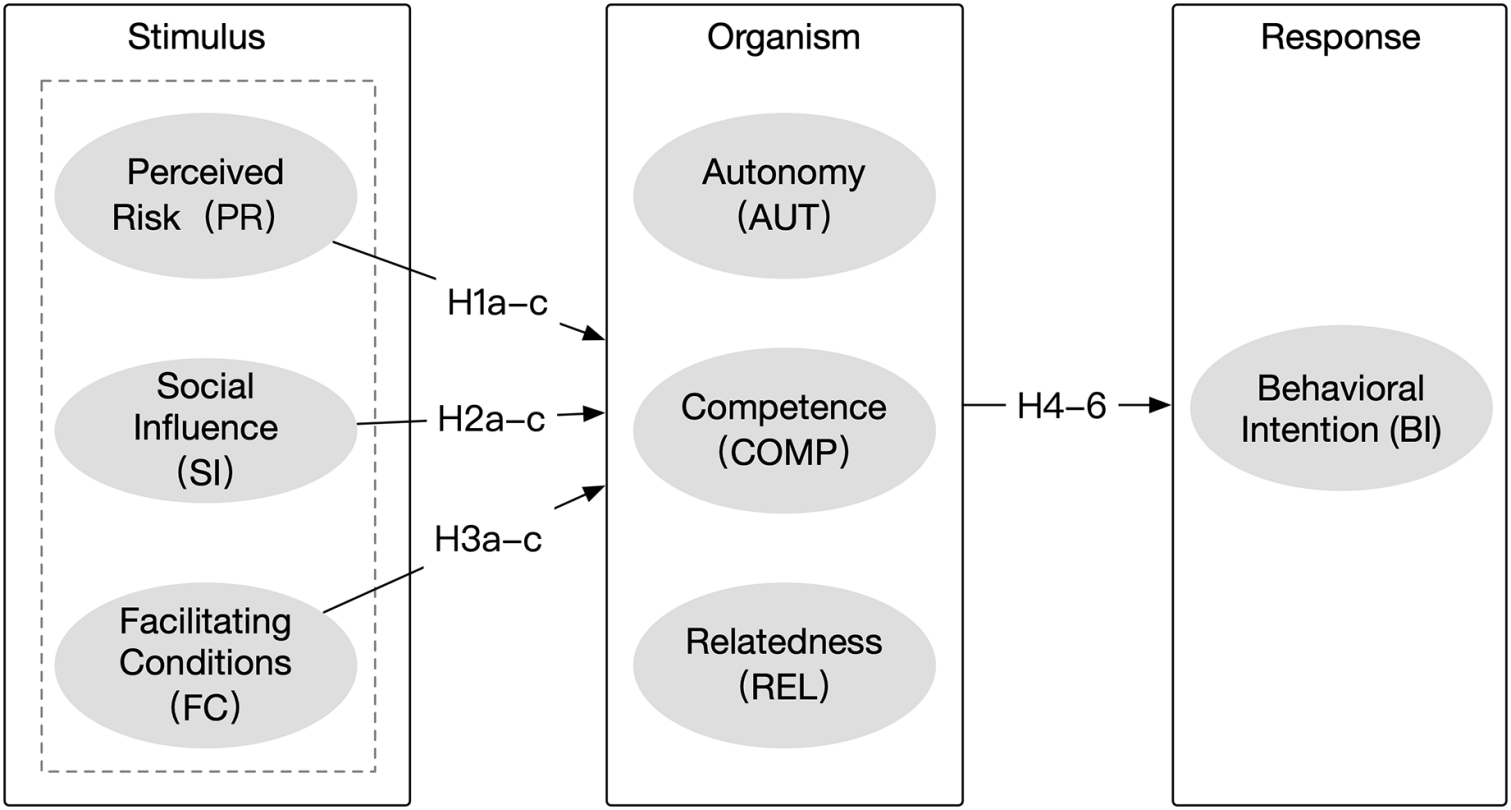
Conceptual model.

Based on the above hypothesized paths, this study constructs the following conceptual model shown in Fig 1. SOR supplies the overall structure; PR, SI, and FC form the stimulus layer; AUT, COMP, and REL form the organismic basic-need-satisfaction layer; and BI forms the response layer. The model therefore contains seven core constructs and twelve hypothesized paths.

## 3 Methods

### 3.1 Research design

This study used a quantitative, cross-sectional questionnaire design to test the theoretical model and hypotheses. The design measured perceived risk, social influence, facilitating conditions, three basic psychological need-satisfaction states, and behavioral intention in the natural work context of fashion-design practitioners. The study followed a deductive empirical approach: theory and prior evidence generated the hypotheses, and PLS-SEM tested the proposed relationships among the latent constructs [36].

### 3.2 Variable measurement and questionnaire design

To establish content validity, the questionnaire followed the scale-development process proposed by Churchill Jr. [37]. Perceived risk was adapted from Li et al. [11]; social influence and facilitating conditions were adapted from Venkatesh et al. [7]. The measurement items for AUT, COMP, and REL were adapted from Jiang et al. (2024) [4]. The research team adapted wording to the AIGC-assisted fashion-design context. The questionnaire contained 21 measurement items, with three reflective items for each construct, and used a seven-point Likert scale from 1 = strongly disagree to 7 = strongly agree. The full item wording and sources are provided in S1 Appendix.

A small pilot test was conducted before finalizing the questionnaire to further improve the validity of the instrument. The pre-survey invited approximately 20 volunteers with a background in the apparel design profession to complete the initial version of the questionnaire; these candidates had similar characteristics to the formal sample but were not included in the data analysis for the main study. The pre-survey collected feedback from the respondents on the difficulty of understanding the questions and the appropriateness of the wording, and the research team modified and improved the questionnaire accordingly. The results of the pre-survey showed that most of the questions were clear and easy to understand, and a few ambiguous expressions were optimized. Therefore, the formal questionnaire was finalized to contain 21 measurement items covering all the aforementioned constructs, with each construct being measured by three questions. Each item was measured on a seven-point Likert scale, i.e., from “1=Strongly Disagree” to “7=Strongly Agree”, and respondents rated each statement according to their own circumstances.

### 3.3 Common method bias

Because all variables were collected through the same self-report questionnaire, common-method bias was possible. The questionnaire clarified anonymity and academic use, separated predictor and outcome items, and avoided presenting all items in a single block. Harman’s one-factor test showed that the first unrotated factor explained 38.2% of the variance, below the 50% heuristic threshold [38]. This result reduces concern about a dominant common factor but does not eliminate all possible common-method bias.

### 3.4 Sample and participants

The respondents were apparel-design practitioners in China, covering groups such as apparel designers working in apparel companies, independent designers, college teachers of related majors, and senior students of apparel design majors. To ensure the professional relevance of the sample, all respondents were required to meet the following criteria: (1) experience in the field of apparel design or practice in the past 6 months; and (2) knowledge of or experience in the use of design tools based on AIGC. This standardized screening helped to improve the validity of the data, as only respondents with real exposure to apparel design and AIGC could provide meaningful answers to the relevant questions.

Before data collection, an a priori G*Power analysis used Cohen’s medium effect size (f^2^ = 0.15), α = 0.05, power = 0.80, and three predictors, corresponding to the maximum number of structural paths directed at any endogenous construct; the minimum required sample size was 77 [39,40]. The final analytical dataset contained 318 valid response records with complete data across the 21 model items; therefore, all 318 records were used in the PLS-SEM analyses.

### 3.5 Data collection procedure

The data collection was mainly carried out during the period of February to March 2025. In order to improve the efficiency of questionnaire distribution and retrieval, this study utilized an online questionnaire, which was released through the Questionnaire Star platform. Online questionnaires have the advantages of low cost, wide coverage, and quick response to effectively reach geographically dispersed target groups [41]. The research team began by explaining the background and purpose of the study to potential respondents on the opening page of the questionnaire, emphasizing that participation was completely voluntary and responses were anonymous, and included screening questions to confirm that respondents met the requirements for experience in the apparel design industry and knowledge of the AIGC.

Participants were first presented with a detailed informed consent statement on the opening page of the questionnaire. They were required to indicate their voluntary participation by selecting a consent checkbox before proceeding. This procedure constituted electronic written informed consent. Only respondents who provided consent were allowed to continue with the survey, while those who declined were automatically exited. All participants were adults (aged 18 years and above), and no minors were involved in this study. This research was conducted in accordance with the principles of the Declaration of Helsinki. Formal ethical approval was not required because the study involved an anonymous online questionnaire, did not collect any personally identifiable or sensitive information, posed no foreseeable risk or harm to participants, and did not involve any intervention or deception. According to Article 32 of the Ethical Review Measures for Life Sciences and Medical Research Involving Humans issued by the National Health Commission of China (2023), studies that meet these conditions may be exempted from ethical review.

Only respondents who met the screening criteria completed the formal questionnaire. The survey link was distributed through design departments in apparel companies, universities with apparel-design programs, social-media channels, and professional designer communities. The final dataset contains 318 complete model questionnaires. Demographic information was available for 312 respondents; six respondents did not provide demographic items but had complete responses to all model items and were retained in the PLS-SEM analysis. Table 1 reports the demographic distributions using the 312 respondents with available demographic information.

**Table 1:** Demographic profile of respondents with available demographic information (n = 312).

Demographic	Category	Frequency	Percentage (%)
Gender	Male	101	32.37
	Female	211	67.63
Age	18-25	101	32.37
	26-35	175	56.09
	36-45	32	10.26
	46 and above	4	1.28
Education level	High School or Below	37	11.86
	Bachelor’s Degree	252	80.77
	Graduate Degree or Higher	23	7.37
Occupation	Independent Designer	22	7.05
	Clothing company designers	152	48.72
	Fashion colleges or research institutes	138	44.23

**Note:** Table 1 uses n = 312 because six respondents did not provide demographic information. All 318 complete model-item records were retained for PLS-SEM.

## 4 Results

SmartPLS version 4.1.0.3 was used to evaluate the measurement model and estimate the structural model using partial least squares structural equation modeling (PLS-SEM).

### 4.1 Measurement model

Before assessing the structural model, the reliability and convergent validity of the measurement model were evaluated (Table 2). Cronbach’s alpha values ranged from 0.828 to 0.933, rho_A values ranged from 0.881 to 0.934, and composite reliability values (rho_C) ranged from 0.893 to 0.957. Standardized indicator loadings ranged from 0.786 to 0.944, with all values exceeding the recommended threshold of 0.70. Average variance extracted (AVE) values ranged from 0.737 to 0.881 and exceeded the recommended threshold of 0.50. These results support the internal consistency reliability and convergent validity of the measurement model [42].

**Table 2:** Results of internal and convergent reliabilities.

Construct	Item	Loading	Cronbach’s α	rho_A	rho_C	AVE
Perceived Risk	PR1	0.912	0.846	0.922	0.904	0.758
	PR2	0.874				
	PR3	0.825				
Social Influence	SI1	0.920	0.839	0.903	0.901	0.754
	SI2	0.786				
	SI3	0.892				
Facilitating Conditions	FC1	0.912	0.828	0.917	0.893	0.737
	FC2	0.810				
	FC3	0.850				
Autonomy	AUT1	0.939	0.918	0.919	0.948	0.860
	AUT2	0.928				
	AUT3	0.914				
Competence	COMP1	0.943	0.933	0.934	0.957	0.881
	COMP2	0.929				
	COMP3	0.944				
Relatedness	REL1	0.899	0.880	0.881	0.926	0.806
	REL2	0.889				
	REL3	0.905				
Behavioral Intention	BI1	0.934	0.930	0.930	0.955	0.877
	BI2	0.938				
	BI3	0.937				

Discriminant validity was further assessed with the Fornell–Larcker criterion (Table 3). The square root of each construct’s AVE exceeded its correlations with the other constructs, supporting discriminant validity and the use of the measurement model in the structural analysis [42].

**Table 3:** Discriminant Validity (Fornell-Larcker).

	AUT	BI	COM	FC	PR	REL	SI
AUT	0.927						
BI	0.702	0.936					
COMP	0.628	0.78	0.939				
FC	0.379	0.333	0.389	0.858			
PR	−0.452	−0.458	−0.551	0.026	0.871		
REL	0.461	0.521	0.463	0.179	−0.315	0.898	
SI	0.333	0.351	0.3	−0.017	−0.097	0.619	0.868

### 4.2 Structural model and hypothesis testing

The structural model was estimated with the 318 complete response records. The R^2^ values for AUT, COMP, and REL were 0.445, 0.531, and 0.487, respectively, while the R^2^ for BI was 0.696. The model therefore explained 69.6% of the variance in behavioral intention. VIF values ranged from 1.001 to 1.758, below the conservative threshold of 3.3, indicating no serious multicollinearity [43].

We tested the significance of the structural paths with 5,000 bootstrap resamples. Table 4 shows that PR negatively predicted AUT (β = −0.433, p < 0.001), COMP (β = −0.537, p < 0.001), and REL (β = −0.263, p < 0.001), supporting H1a–H1c. SI positively predicted AUT (β = 0.298, p < 0.001), COMP (β = 0.255, p < 0.001), and REL (β = 0.597, p < 0.001), supporting H2a–H2c. FC positively predicted AUT (β = 0.395, p < 0.001), COMP (β = 0.407, p < 0.001), and REL (β = 0.196, p < 0.001), supporting H3a–H3c.

**Table 4:** Structural model.

Hypotheses	Path	Std.beta	T statistics
H1a	PR → AUT	−0.433^***^	11.348
H1b	PR→COMP	−0.537^***^	16.278
H1c	PR → REL	−0.263^***^	6.455
H2a	SI → AUT	0.298^***^	7.711
H2b	SI→COMP	0.255^***^	5.909
H2c	SI → REL	0.597^***^	16.286
H3a	FC → AUT	0.395^***^	11.200
H3b	FC→COMP	0.407^***^	11.282
H3c	FC → REL	0.196^***^	5.009
H4	AUT → BI	0.313^***^	7.487
H5	COMP→BI	0.520^***^	13.471
H6	REL → BI	0.136^***^	3.839

**Note.** ***p < 0.001; **p < 0.01; *p < 0.05. Path significance was assessed using 5,000 bootstrap resamples.

**Fig 2:**
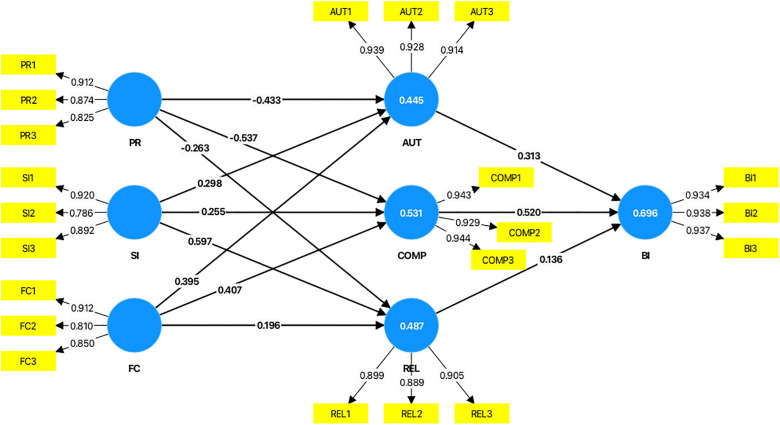
Structural model path coefficient diagram (*R²* in circles).

Among the stimulus-to-organism paths, PR had the strongest negative association with COMP (β = −0.537***), whereas SI had the strongest positive association with REL (β = 0.597***). AUT (β = 0.313***), COMP (β = 0.520***), and REL (β = 0.136***) each positively predicted BI, supporting H4–H6. The complete structural model, including the standardized path coefficients, indicator loadings, and R^2^ values of the endogenous constructs, is presented in Fig 2. The specific indirect effects are reported in Table 5. Every 95% bootstrap confidence interval excluded zero, confirming transmission from each stimulus to BI through each basic psychological need.

**Table 5:** Specific indirect effects of external stimuli on behavioral intention through basic psychological needs.

Path	Indirect effect (β)	Bootstrapped 95% CI	Inference
PR → AUT → BI	−0.135	[−0.177, −0.096]	Significant
SI → AUT → BI	0.093	[0.063, 0.125]	Significant
FC → AUT → BI	0.124	[0.087, 0.163]	Significant
PR → COMP → BI	−0.279	[−0.335, −0.225]	Significant
SI → COMP → BI	0.133	[0.088, 0.180]	Significant
FC → COMP → BI	0.211	[0.165, 0.261]	Significant
PR → REL → BI	−0.036	[−0.059, −0.016]	Significant
SI → REL → BI	0.081	[0.038, 0.124]	Significant
FC → REL → BI	0.027	[0.011, 0.046]	Significant

**Note:** Indirect effects were estimated with the same 5,000-resample bootstrap procedure used for the structural paths. All confidence intervals exclude zero.

To further evaluate explanatory power and predictive relevance, Stone–Geisser’s Q^2^ values and f^2^ effect sizes were examined for the endogenous constructs and key structural paths. The effects of PR on COMP (f^2^ = 0.609), SI on REL (f^2^ = 0.689), COMP on BI (f^2^ = 0.505), and FC on COMP (f^2^ = 0.352) met or slightly exceeded the threshold for a large effect. The effect of PR on AUT (f^2^ = 0.335) was medium [44]. These results further support the model’s explanatory strength.

All Q^2^ values were greater than zero, with values of 0.604 for BI, 0.460 for COMP, 0.378 for AUT, and 0.387 for REL, indicating predictive relevance [44]. The comparatively high Q^2^ values for BI and COMP suggest particularly strong predictive relevance for these endogenous constructs. Collectively, the path coefficients, R^2^ values, effect sizes, and Q^2^ values indicate satisfactory explanatory and predictive performance.

## 5 Discussion and conclusion

The SOR model shows that perceived risk, social influence, and facilitating conditions shape Chinese fashion designers’ AIGC adoption intention through distinct organismic states. PR reduced autonomy, competence, and relatedness, whereas SI and FC supported these states. These findings extend prior work by showing how risk, social norms, and resources enter the psychological process of technology adoption in fashion design.

At the organismic level, this study tested the three SDT basic psychological needs as separate constructs. AUT, COMP, and REL each positively predicted BI, so designers who felt more self-directed, capable, and connected reported stronger adoption intention. Because intrinsic motivation was not measured directly, the results do not establish that PR reduces intrinsic motivation or that SI and FC increase it; they establish the more specific pathway from contextual stimuli to need satisfaction and then to BI.

Competence had the strongest association with BI (β = 0.520, p < 0.001). Fashion designers must translate prompts into usable concepts, evaluate multiple outputs, control style and garment details, and integrate selected outputs into professional workflows. These tasks require practical tool mastery and confidence in making design judgments, so competence may be the most immediate condition for treating AIGC as a controllable collaborator rather than an unpredictable generator. The result is consistent with SDT’s emphasis on competence as a basis for sustained engagement [34] and with evidence that designers’ self-determination experiences shape their responses to AI participation [4].

In summary, the model identifies two complementary routes to AIGC adoption: perceived risk frustrates basic psychological need satisfaction, while social influence and facilitating conditions support it. The significant specific indirect effects show that the SOR sequence is empirically supported in this sample. The model should therefore be interpreted as a need-satisfaction mediation model, not as a direct measurement model of intrinsic motivation.

### 5.1 Comparison with related studies

To address possible overlap, we explicitly compared the present study with two related publications. Yang and Jin [45] analyzed 267 valid responses obtained from 335 collected questionnaires from Chinese fashion designers recruited through online design communities, professional associations, and social-media groups. The published article did not report the exact fieldwork dates. That study examined how personalization fit, perceived content quality, industry pressure, and perceived technological risk affected adoption intention through self-efficacy, innovativeness, and task–technology fit. Yang and Jin (2026) [5] collected 352 valid responses from January to February 2025 through the Questionnaire Star platform, recruiting participants through apparel companies and colleges and universities; it tested an SDT–TAM–perceived-risk model in which autonomy, competence, and relatedness affected perceived ease of use and perceived usefulness, which then predicted behavioral intention. By contrast, the present survey was separately administered in February–March 2025 using a different questionnaire and an independently exported 318-record analytical dataset. It tests whether perceived risk, social influence, and facilitating conditions affect behavioral intention through autonomy, competence, and relatedness within an SOR process. No response record from either published dataset was pooled with or reused in the present analysis. Because the three surveys were anonymous, participant-level identity matching was not possible. We therefore cannot exclude the possibility that some individuals may have independently participated in more than one survey.

### 5.2 Theoretical implications

- This study integrates SOR with SDT by assigning each theory a distinct explanatory role. SOR specifies how external stimuli become organismic states and behavioral responses. SDT specifies the organismic states as autonomy, competence, and relatedness. Perceived-risk research motivates the inhibitory stimulus, while UTAUT motivates the social and resource-related stimuli. This bounded integration avoids treating risk, social influence, facilitating conditions, and psychological needs as interchangeable predictors and provides a clearer rationale for each hypothesized pathway.
- The results extend SOR-based technology-acceptance research by testing both a negative stimulus and enabling stimuli in the same fashion-design model. PR was associated with lower need satisfaction, whereas SI and FC were associated with higher need satisfaction. The nine significant specific indirect effects further show how these external conditions are transmitted through AUT, COMP, and REL to BI. This evidence supports the use of SOR as a process model while preserving the conceptual distinction between SDT needs and intrinsic motivation.
- The study also provides a contextualized account of AIGC adoption among Chinese fashion-design practitioners. Unlike generic technology-use models, the present model captures authorship and control through AUT, tool mastery through COMP, and professional community through REL. The strongest organismic path was COMP→BI, highlighting the importance of designers’ practical ability to direct, evaluate, and refine AIGC outputs in real design work.

### 5.3 Practical implications

The findings of this study have important practical implications for the popularization and application of AIGC in the field of creative design.

- Perceived risk may hinder fashion designers from adopting AIGC technology, so all stakeholders should work to alleviate fashion designers’ concerns about risks and uncertainties. AIGC service providers and industry organizations can enhance the transparency and credibility of the tools by explaining matters such as copyright ownership, security, and privacy, thereby alleviating designers’ fears of unknown risks. For fashion design specifically, this includes clarifying ownership of AIGC-generated sketches and patterns, disclosing the training-data sources behind style generation to reduce the risk of inadvertent plagiarism of other designers’ work, and providing provenance or watermarking features so designers can document their own creative contribution for portfolio and rights-management purposes. This recommendation is consistent with Wang and Chen [19]. Companies should also emphasize that AIGC serves as a creative aid rather than a replacement for designers. In addition, offering targeted training and support can improve designers’ confidence and reduce insecurity. Prior studies have shown that privacy safeguards and learning resources are effective in lowering skepticism and increasing adoption rates [2]. As designers become more proficient with AIGC, their perceived risk tends to decline, increasing their willingness to adopt.
- This study emphasizes the importance of supporting designers’ autonomy, competence, and relatedness during AIGC adoption. Organizations should preserve designers’ control over creative briefs, generation parameters, output selection, revision, and final approval; provide scaffolded training and feedback to strengthen competence; and create peer-learning communities and collaborative review routines to support relatedness. These conditions allow designers to experience AIGC as a tool they can direct and master rather than as an imposed system that threatens authorship or professional identity.
- Because AIGC use in fashion design is largely prompt-driven, design firms and fashion-education programs should build structured prompt-engineering training that covers fabric, silhouette, and trend-specific vocabulary so designers can translate creative intent into usable outputs efficiently, rather than relying on trial-and-error. Workflow design should also formalize the designer-AI division of labor: designers set the creative brief, curate and iterate on AIGC outputs, and perform the final aesthetic and technical review, so that AIGC functions as a controllable collaborator embedded in professional practice rather than an autonomous decision-maker. Design schools and companies can further support this collaboration by building shared prompt libraries and case repositories that let designers learn from one another's successful AIGC workflows.
- External incentives can be appropriately used to increase adoption rates, such as through industry awards or promoting designers who have used AIGC technology to produce outstanding design results, thereby creating a positive demonstration effect. When external motivation is internalized by individuals, it can also have a positive impact on behavior. Therefore, managers should focus on combining organizational goals with the personal values of designers to help them transform external requirements for using AIGC into internal recognition.

### 5.4 Limitations and future research

This study has four main limitations. First, the model measures autonomy, competence, and relatedness as basic psychological need-satisfaction constructs but does not directly measure intrinsic or extrinsic motivation; future studies should include validated motivation scales to test those mechanisms explicitly. Second, the cross-sectional design measures behavioral intention at one time point and cannot establish how intention develops into sustained use. Longitudinal or behavioral-log studies are needed. Third, the Chinese sample and the missing demographic responses for six participants limit generalizability and subgroup analysis. Fourth, the anonymous recruitment design prevents participant-level matching across related surveys; future multi-study programs should use ethically appropriate, non-identifying study codes to document overlap without compromising privacy.

Future research should compare designers across countries, organizational settings, and experience levels, and should examine whether copyright governance, prompt training, and collaborative tool design moderate the paths identified here. Experimental or longitudinal designs could also test whether gains in competence precede increases in actual AIGC use.

## Supporting information

S1 Appendix Measurement items and sources. (DOCX)

S1 Data De-identified item-level survey data used in the PLS-SEM analysis. (XLSX)
