# Supplementary material for: Exploring fashion designers’ acceptance of AIGC: A dual-pathway analysis from the stimulus–organism–response perspective
Source: PLoS One. 2026 Aug 17;21(8):e0356536. doi: 10.1371/journal.pone.0356536 (PMC13480654; doi:10.1371/journal.pone.0356536)
Supplement: Supplementary file 1 [file pone.0356536.s001.docx]

**Appendix A**

| **Construct** | **Item** | **References** |
| --- | --- | --- |
| Perceived Risks | PR1: I think that using AIGC may involve risks to originality, such as generating designs that are similar or identical to those of other designers. | (Li et al., 2024) |
|  | PR2: I think that using AIGC may involve risks to copyrights, such as disputes over intellectual property rights with other designers or companies. |  |
|  | PR3: I think that using AIGC in fashion design may reduce my professional value because it may make my design skills more easily replaced. |  |
| Social Influence | SI1: I am surrounded by key colleagues who think I should try to create with AIGC tools. | (Venkatesh et al., 2012) |
|  | SI2: AIGC tools are generally encouraged in my design circle. |  |
|  | SI3: If I don't use AIGC tools, I will feel left behind in the industry. |  |
| Facilitating Condition | FC1: My work environment provides the technical support needed to use AIGC (e.g., hardware/software configurations) | (Venkatesh et al., 2012) |
|  | FC2: I have easy access to help or how-to guides for using the AIGC tool. |  |
|  | FC3: I have someone who can help when I have problems using AIGC (e.g., a coworker or a technician). |  |
| Autonomy | AUT1: I can make creative decisions independently when designing with generative AI. | (Jiang et al., 2024) |
|  | AUT2: I can express my creativity and style freely when designing with generative. |  |
|  | AUT3: When designing with generative AI, I do not feel constrained by technology. design, I do not feel guided by technical constraints or external influences. |  |
| Competence | COM1: I am confident in my skills and abilities to use AI. | (Jiang et al., 2024) |
|  | COM2: I feel that generative AI helps to improve my drawing skills and creativity. |  |
|  | COM3: I feel a sense of creative fulfillment when using generative AI. |  |
| Relatedness | REL1: Designers using generative AI are willing to collaborate and discuss ideas. | (Jiang et al., 2024) |
|  | REL2: Designers using generative AI are willing to share their experiences and skills to build social connections. |  |
|  | REL3: I feel a sense of belonging and connection to a community of designers when using AIGC. |  |
| Behavior Intention to Use | BI1: I plan to use AIGC tools in my fashion design work. | (Venkatesh et al., 2003) |
|  | BI2: I plan to incorporate AIGC into my design process in the future. |  |
|  | BI3. I predict that I will use AIGC tools regularly in my design projects. |  |
